# Practical Observations on Amputation, and the After-Treatment; to Which Is Added, an Account of the Amputation above the Ancle with a Flap

**Published:** 1783

**Authors:** 


V. *Practical Obſervations on Amputation, and the after-treatment; to which is added, an account of the Amputation above the ancle with a flap. The whole illuſtrated by caſes. By* Edward Alanſon, *Surgeon to the Liverpool Infirmary.* The 2d edition, greatly enlarged, 8vo. Johnſon, *London*, 1782. 296 pages. 5s.

MR. Alanſon's deviations from the uſual practice conſiſt in the mode of dividing the parts, and in the after-treatment. Firſt, as to the operation, he differs in the application of the tape, the quantity of ſkin ſaved, and the manner of executing the double inciſion. As every moment's delay after the tourniquet is applied, detains the patient in a painful ſtate of mind, he univerſally omits the tape; but in ſome few inſtances, as in caſes of abſceſſes above the knees, where ſuch a guide has been deemed particularly uſeful, he marks out a circular line

 with with ink or ſome coloured fluid, and cuts with a les knife.

Before the circular inciſion is begun, an aſſiſtant graſps the limb circularly with both hands and firmly draws up the ſkin and muſcles, by which means the operator's knife paſſes through the integuments with conſiderable facility and diſpatch.

After the inciſion is made through the ſkin and adipoſe membrane, the aſſiſtant ſtill continuing a ſteady ſupport of the parts, the cellular and ligamentous attachments are to be divided with the point of the knife till as much ſkin is drawn up as will, with the united aſſiſtance of the particular diviſion of the muſcles hereafter recommended, fully cover the whole ſurface of the wound with the moſt perfect eaſe.

Inſtead of dividing the muſcles in a perpendicular manner down to the bone, the knife is directed to be carried obliquely upwards, ſo as to lay it bare (in the thigh for inſtance) three or four inches higher than is uſually done. The retractor is then to be applied, and the bone denuded of its perioſteum where the ſaw is to paſs. "A ſtump, formed in the thigh, agreeably to "this plan—ſays our ingenious author—may

"in

" in fome degree be faid to refemble a conical
" cavity, and the parts thus divided are obvi-
" oufly the beft calculated to prevent a fugar-
" loaf ftump."

The arteries are to be drawn out with the tenaculum, and tied with a flender ligature as naked as poffible, cutting off the ligature much longer than ufual, otherwife they will afterwards be drawn within the edges of the wound.

The fkin is to be brought forwards immediately after the operation, and fupported in a fixed pofition by means of a bandage made of the fineft Welch flannel, which being foft and elaftic, eafily yields to the inflammatory tenfion.

Before the wound is dreffed, its whole furface is to be carefully examined, to fee that no veffel has efcaped the eye of the operator. The author has frequently perceived a pulfation where no hæmorrhage has previoufly appeared, and turned out a fmall clot of blood from within an artery of confiderable fize.

The furface of the wound is now to be well cleaned with a fpunge and warm water, and the fkin and mufcles are to be gently brought forwards, and fupported by the flannel roller, which, after being paffed round the body, is to be carried two or three times rather tight round the upper part of the thigh, and afterwards brought forwards in a circular direction to the extremity of the ſtump, not ſo tight as to preſs rudely or forcibly, but to give an eaſy ſupport to the parts. This circular roller, however, is not to be too long continued, leſt the thigh ſhould emaciate under the preſſure it occaſions. The ſkin is to be ſecured by ſlips of linen or lint ſpread with cerate, and over them a ſoft tow pledget. The whole is to be retained with the many-tailed bandage.

The ſtump is not to be raiſed with pillows, as this draws back the poſterior muſcles, but it is to be elevated merely half a hand's breadth, by which means the muſcles are put into an eaſy relaxed poſition.

Our author objects to the application of lint to the ſurface of the wound, becauſe it irritates, increaſes the diſcharge, renders the pus acrimonious by retaining it, and thus excites hectic fever. It likewiſe, he adds, promotes ſpaſm, dilates the whole ſurface of the wound, conduces to promote hæmorrhage, and is often the principal cauſe of exfoliation.—During the ſuppurative ſtage, he generally gives the bark, and is careful to obviate coſtiveneſs.

Such is the method recommended by Mr. Alanfon, and he affures us, that fince he has practifed it, he has met with only one cafe where there occurred the fmalleft exfoliation. As the cicatrix is fo fmall, viz. only a fingle line drawn acrofs the ftump from fide to fide, the parts are fooner capable of bearing the preffure of a wooden leg, and the bone being covered with a large flap of old fkin and mufcular fubftance, the wound is lefs liable to break out again. He has operated in thirty-five cafes, fuch as promifcuoufly occurred at the Liverpool infirmary, without the lofs of a fingle patient. The fymptomatic fever, fpafm, and difcharge have in all been flight. There has not been a neceffity to remove the dreffings on account of hæmorrhage in a fingle inftance, and at the end of a month the wound has either been perfectly healed, or lefs than a fixpenny piece. Whereas before this improved plan was adopted, out of forty-fix amputations, at which Mr. Alanfon was prefent, in hofpital and private practice, ten died; one of a locked jaw, two of hæmorrhage from the whole furface of the ftump, four of hectic fever and extenfive fuppuration, and three from a fpreading gangrene on the furface of the ftump, eighteen had an hæmorrhage, fix from the whole furfurface of the wound, and twelve from one or more particular veffels. In nearly the whole, the fymptomatic fever was violent; the ftartings frequent; the fuppuration large; the furface of the wound extenfive; and in all, the firft dreffings were painful. In moft of them there was an exfoliation; in feveral a fugar-loaf ftump; and in fome, the wound remained incurable.

We are next prefented with fome very judicious mifcellaneous obfervations on amputation, and the air of hofpitals, in which the author points out the difadvantages arifing from crowded infirmaries, and propofes many excellent regulations relative to their management. No ward, he obferves, fhould be inhabited for more than four months together, and the walls fhould then be fcraped and white-wafhed.—He recommends iron bedfteads, frequent change of bedding, and its daily expofure to the open air. All incurable or infectious cafes, he thinks, fhould be refufed admittance, and that amongft thefe fhould be claffed old chronic ulcers of the legs.

This paper is followed by obfervations on the amputation with a flap, above the ancle, in which feveral cafes are related, with remarks in proof of its utility. Mr. Alanfon treats likewife wiſe of what is called union by the firſt intention, and of the exfoliation of cartilages. He relates a very intereſting account of an amputation of the arm at its articulation with the ſcapula, and the work cloſes with ſeveral hiſtories and caſes in proof of the doctrines advanced in the preceding parts of it, but for theſe, and a variety of other uſeful matter, we muſt refer our readers to the volume itſelf.

